# Signal Detection Method for OTFS System Based on Adaptive Wavelet Convolutional Neural Network

**DOI:** 10.3390/s26041397

**Published:** 2026-02-23

**Authors:** You Wu, Mengyao Zhou

**Affiliations:** Ocean College, Jiangsu University of Science and Technology, Zhenjiang 212000, China; 231212201104@stu.just.edu.cn

**Keywords:** orthogonal time–frequency space, conventional convolutional neural network, adaptive wavelet convolutional module, signal detection

## Abstract

In Orthogonal Time–Frequency Space (OTFS) systems, signal detection algorithms based on convolutional neural networks (CNNs) suffer from insufficient feature extraction and are limited by local mixing. Additionally, fixed convolution kernels struggle to match the sparsity and non-stationary characteristics of OTFS signals in the delay-Doppler domain, resulting in slow convergence and high training costs. We do not stop at simply integrating more features outside the existing CNN framework. Instead, we go deeper into the network and replace the fixed convolution kernels with wavelet convolution layers that have time–frequency-adaptive capabilities. This fundamental change allows the network to more intrinsically match the physical characteristics of OTFS signals in the delay-Doppler domain, thereby achieving excellent detection performance while also gaining faster convergence efficiency. Therefore, this paper proposes a signal detection method using an adaptive wavelet convolutional neural network (AWCNN). The approach replaces the first convolutional layer of a standard CNN with an adaptive wavelet layer, which leverages the time–frequency localization properties of Sym4 wavelet kernels along with learnable scaling and translation factors. This enhances the network’s ability to extract sparse features from OTFS signals. Additionally, the model incorporates both the original received signal and preliminary estimates from the message-passing (MP) algorithm as input features, enriching the dataset and further improving detection performance. Experimental results demonstrate that the AWCNN model achieves superior convergence efficiency compared to the CNN model, which attains a bit error rate (BER) comparable to that of the CNN algorithm at a low signal-to-noise ratio of 2 dB, operating without the need for pilot-assisted channel state information acquisition.

## 1. Introduction

In recent years, in high-speed communication scenarios [1], it has been determined that severe Doppler spread can destroy the orthogonality among Orthogonal Frequency Division Multiplexing (OFDM) subcarriers, leading to intercarrier interference (ICI) and consequently degrading communication performance. As a result, Orthogonal Time–Frequency Space (OTFS) technology was proposed by Hadani et al. [2]. As a key candidate technology for sixth-generation (6G) mobile communication systems [3,4,5,6], it aims to overcome the limitations of OFDM in high-mobility scenarios. In OTFS technology, the data symbols at the transmitter and receiver are all mapped onto a two-dimensional delay-Doppler (DD) domain, so that each data symbol carried in the DD domain is spread across the entire time–frequency plane. This allows for full time–frequency domain diversity gain from the channel; the DD domain equivalent channel exhibits good sparsity. Therefore, fully leveraging these advantages can reduce the complexity of signal detection algorithms and has significant practical value.

Regarding signal detection in OTFS systems, linear detection algorithms such as Zero Forcing (ZF) [7] and Minimum Mean Square Error (MMSE) [8] are straightforward to implement but involve computationally intensive matrix inversion operations. On the other hand, nonlinear algorithms like Maximum Likelihood (ML) [9] and Maximal Ratio Combining (MRC) [10] offer theoretically optimal performance but are hindered by prohibitively high computational complexity, limiting their practical applicability. Raviteja et al. introduced a message-passing (MP) [11] algorithm that exploits the inherent sparsity of OTFS channels. By iteratively updating and selecting the symbol with the maximum posterior probability as the detection result, the MP algorithm effectively mitigates the impact of Doppler spread. While improved versions of MP perform well in strongly sparse channels, they still face challenges of high computational complexity in multipath-rich environments as the number of iterations increases.

Meanwhile, deep learning has achieved rapid development in communication systems owing to its powerful capabilities in feature learning and representation. Ye H et al. [12] first introduced the integration of Deep Neural Networks (DNNs) with OFDM to perform channel estimation and signal detection. Li Q et al. [13,14] proposed a DNN-based OTFS receiver scheme, which replaces traditional channel estimation and signal detection modules with DNN, effectively overcoming the issues of high computational complexity and high bit error rate in conventional detection algorithms. Zhou Shuo et al. [15] introduced a signal detection method based on residual networks, which transforms input data into the DD domain and concatenates the real and imaginary parts of signals into multi-dimensional representations to maximize the utilization of critical information, achieving low-bit-error-rate performance. Y. K. Enku et al. [16] combined traditional algorithms with deep learning, using the MP algorithm for data augmentation to enrich the dataset and enhance the performance of a convolutional neural network (CNN). Although CNN architectures can effectively capture spatial correlations in DD domain signals, CNN models are complex to train, prone to overfitting due to limited local mixing, and exhibit reduced generalization ability.

CNN-based detection algorithms [17] suffer from insufficient feature extraction and limitations in local mixing. Moreover, their fixed convolutional kernels struggle to match the sparsity and non-stationary characteristics of OTFS signals in the DD domain, resulting in slow convergence and high training costs. To address these limitations, some studies have attempted to increase the convolutional kernel size in CNNs, but such improvements quickly reach a saturation point. In response, this paper proposes a signal detection method based on an adaptive wavelet convolutional neural network. By leveraging the time–frequency localization properties of Sym4 wavelet basis functions and incorporating learnable scaling and translation factors, the proposed method replaces the first convolutional layer of a standard CNN with an optimized adaptive wavelet convolutional module. This enhancement improves the extraction of sparse features in OTFS signals. Additionally, the model takes as input both the original received signal and the preliminary estimates from the MP algorithm, further boosting detection performance.

## 2. Materials and Methods

### 2.1. OTFS System Model

**Figure 1:**
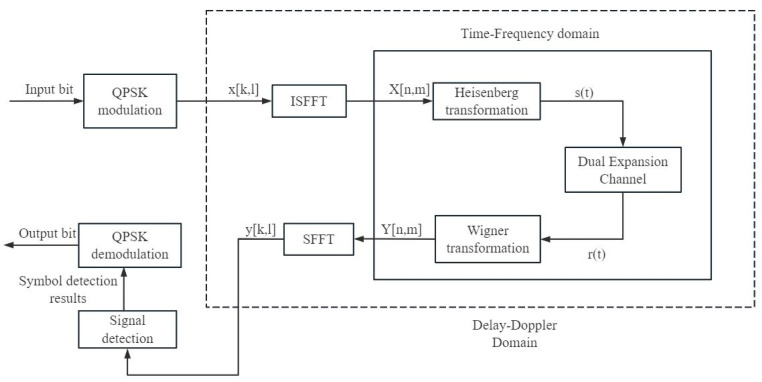
OTFS modem block diagram.

OTFS proposes a two-dimensional modulation technology in the DD domain. Through two-dimensional transformation, the two-dispersion channels of time and frequency can be converted into the DD domain to become an approximately non-fading channel [18]. The block diagram of the modulation and demodulation technology of this system is shown in Figure 1.

#### 2.1.1. Transmitter

In an OTFS system, let N be the number of subcarriers and M the number of OTFS symbols, without loss of generality. QPSK modulation is adopted as a representative case in this work to validate the proposed AWCNN-based detection scheme. The proposed method is modulation-agnostic and can be naturally extended to higher-order modulations such as 16QAM and 64QAM. Due to the scope and space limitations, performance evaluation under higher-order modulations is left for future work. The modulated symbols form an M × N matrix of data symbols, which is then mapped onto the DD domain grid x [k, l], the inverse symplectic finite Fourier transform (ISFFT), and these DD domain symbols are transformed into time–frequency domain symbols X [n, m], populating the time–frequency signal grid. This transformation is mathematically formulated in (1):

(1) $$X\left[n,m\right]=\frac{1}{\sqrt{MN}}\sum_{K=0}^{N-1}\sum_{l=0}^{M-1}x\left[k,l\right]e^{-j2\pi \left(\frac{nk}{N},\frac{ml}{M}\right)}$$

After obtaining discrete time–frequency domain symbols X [n, m], the Heisenberg transformation is used to convert X [n, m] into a continuous time-domain transmission signal, as expressed by Formula (2):

(2) $$s\left(t\right)=\sum_{n=0}^{N-1}\sum_{m=0}^{M-1}X\left[n,m\right]g_{tx}\left(t-nT\right)e^{j2\pi m\Delta f\left(t-nT\right)}$$

$g_{\mathrm{tx}}\left(t\right)$ represents the pulse-shaping waveform at the OTFS transmitter. When $g_{\mathrm{tx}}\left(t\right)$ is a rectangular pulse waveform, the Heisenberg transformation becomes the traditional discrete Fourier and the inverse discrete Fourier transform.

#### 2.1.2. Dual Expansion Channel

In doubly dispersive channels, the channel is time-varying. The time-domain impulse response of the channel is expressed by Equation (3):

(3) $$h(\tau ,v)=\sum_{P=1}^{P}h_{p}\delta (\tau -\tau_{p})\delta (v-v_{p})$$

where $h_{p},\tau_{p},\nu_{p}$ denote the channel gain, delay, and Doppler shift respectively, and p represents the number of paths. The channel gain $h_{p}$ is a complex random variable, which follows a Rayleigh distribution with a uniformly distributed phase. The gains of all paths are normalized, with the normalized power distribution being [1/3, 1/3, 1/3]. The delays and Doppler shifts are discretized as $\tau_{p}=l_{p}/(M\Delta f),v_{p}={\tilde{k}}_{p}+k_{p}/(NT)$, where $l_{p}$ and $k_{p}$ represent integer multiples of delay taps and Doppler taps, respectively, and ${\tilde{k}}_{p}\in [-1/2,1/2]$ denotes the fractional Doppler shift. To avoid inter-symbol interference, a cyclic prefix (CP) is inserted; the CP length should be greater than the maximum delay $L>\tau_{\max}M\Delta f$.

#### 2.1.3. Receiver

After the sending signal passes through the wireless channel, the received signal r(t) of the OTFS system is obtained, as expressed by Formula (4):

(4) $$r\left(t\right)=\int_{v}\int_{\tau }h\left(\tau ,v\right)s\left(t-\tau \right)e^{j2\pi v\left(t-\tau \right)}d\tau dv+u\left(t\right)$$

At the receiving end, u(t) represents the noise. The receiving-end pulse-shaping waveform $g_{\mathrm{tx}}\left(t\right)$ can be used for matching filtering, then the received data can be obtained. For example, Formula (5) indicates the time–frequency domain received signal:

(5) $$Y\left(t,f\right)=A_{g_{rx},y}\left(t,f\right)=\int g_{rx}^{*}\left(t^{\prime }-t\right)r\left(t^{\prime }\right)e^{-j2\pi f\left(t^{\prime }-t\right)}dt^{\prime }$$

Then, sample t=nT,f=mΔf is an interval used to obtain discrete time–frequency domain reception signals. This step is called the Wigner transform, as expressed by Formula (6):

(6) $$Y\left[n,m\right]=A_{g_{rx},y}\left(t,f\right){|}_{t=nT,f=m\Delta f}$$

The time–frequency domain reception signal Y [n, m] is processed using SFFT to obtain the received signal in the time-delay-Doppler domain, as expressed in Formula (7):

(7) $$y\left[k,l\right]=\frac{1}{\sqrt{MN}}\sum_{n=0}^{N-1}\sum_{m=0}^{M-1}Y\left[n,m\right]e^{-j2\pi \left(\frac{nk}{N}-\frac{ml}{M}\right)}$$

According to the derivation of the signal processing flow of the sending and receiving ends of the OTFS system, the input and output relationship of the signal in the DD domain is shown in Formula (8):

(8) Y=HX+G

where $Y,X\in {\mathbb{C}}^{N\times M}$, H is a multiple cycle matrix of NM×NM for describing the channel, and $G\in {\mathbb{C}}^{N\times M}$ is additive Gaussian white noise.

The received signal $y\left[k,l\right]$ undergoes signal detection to obtain symbol detection results and then QPSK demodulation to finally obtain output bits. However, traditional signal detection methods usually rely on mathematical models and specific detection algorithms. With the development of artificial intelligence technology, neural networks, as an important branch of AI, can be combined with OTFS technology to better perform signal detection. To implement CNN-based signal detection in OTFS systems, the input–output relationship of Equation (8) is transformed into a representation suitable for real-valued convolution. In many deep learning methods, the imaginary part of complex vectors is often ignored, or the real and imaginary parts are concatenated to form a real-valued vector, which may result in the loss of phase information in the input data, significantly hindering signal detection performance. In this paper, we represent each OTFS frame as two real-valued matrices and stack them to form a three-dimensional tensor, as shown in (9):

(9) $$Y=\left[\begin{array}{l} R(Y)\\ I(Y) \end{array}\right]\;X=\left[\begin{array}{l} R(X)\\ I(X) \end{array}\right]$$

where $Y\in {\mathbb{R}}^{N\times M\times C}$ and $X\in {\mathbb{R}}^{N\times N\times C}$ are the real received signal and the transmitting sign tensor, respectively, and C is called the number of channels.

### 2.2. Neural Network-Based OTFS System Signal Detection Method

This method is based on a convolutional neural network [19] framework and designs an adaptive wavelet convolution module (AWCM). It utilizes the scale and translation factors of the Smy4 wavelet, which are updated iteratively based on model training gradients, replacing the first convolutional layer of the convolutional neural network with the optimized adaptive wavelet convolution module. An AWCM is constructed and its performance is analyzed in the OTFS system signal detection scenario when comparing it with the convolutional neural network model.

#### 2.2.1. CNN-OTFS Network Structure

**Figure 2:**
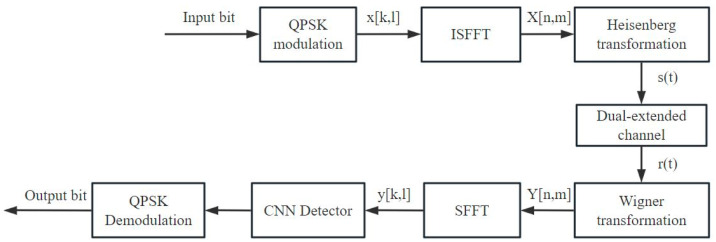
CNN-OTFS system process.

In the signal detection scheme of CNN-OTFS, the symbols transmitted x[k,l] by OTFS in the DD domain are transformed into time–frequency domain signals X[n,m] through ISFFT, then s(t) are transformed into time-domain transmitted signals via the Heisenberg transform. After transmission through a doubly dispersive channel h(τ,v), the received time-domain signals are transformed into time–frequency domain signals at the receiver r(t) via the Wigner transform. The signals Y[n,m] are then transformed into DD domain received signals y[k,l] through SFFT, which are fed into the CNN for signal detection. The output of the CNN is the detected transmitted signal $\hat{x}$. The CNN-OTFS system process is shown in Figure 2.

**Figure 3:**
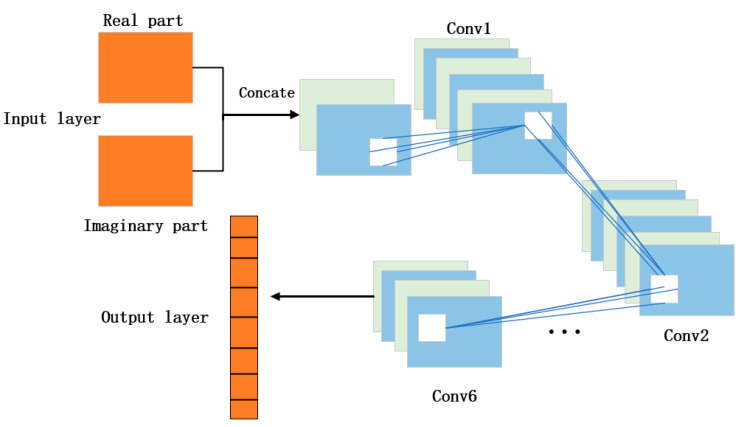
CNN-OTFS structure.

CNN-OTFS takes a two-dimensional signal as input; it first splits it into real and imaginary parts and then feeds them into multiple convolutional layers for training. Since OTFS sparsity depends on precise location information, pooling layers would blur these exact tap positions, leading to a decrease in detection performance. Therefore, the model does not include pooling layers. The CNN-OTFS structure is shown in Figure 3, with each module sequentially containing a two-dimensional convolutional layer, a nonlinear activation layer, and a batch normalization layer. Each module uses ReLU as the activation function to improve training efficiency.

#### 2.2.2. Improved Methods

Traditional wavelet transform [20] relies on predefined and fixed wavelet basis functions whose scaling and translation factors are determined prior to analysis and lack adaptability to specific tasks and data distributions [21]. To address this limitation, this paper employs a learnable Sym4 wavelet to extract multi-scale sparse features from input signals, replacing the fixed-kernel feature extraction approach of conventional convolutional layers. After adapting the translation and scaling factors of the Sym4 wavelet to be learnable parameters, AWCM is introduced as the first layer of the model. During backpropagation in the convolutional network, the weight parameters equivalent to the traditional convolutional kernel are iteratively updated by an adaptive learning rate optimization algorithm. The iteratively updated translation and scaling factors are given by Equations (10) and (11):

(10) $$\left\{\begin{array}{l} \delta_{d_{k}}=\frac{\partial H}{\partial d_{k}}=\frac{\partial H}{\partial z_{k}}\frac{\partial z_{k}}{\partial h_{k}}\frac{\partial h_{k}}{\partial \psi_{d,s_{1},s_{2},\theta }^{k}}\frac{\partial \psi_{d,s_{1},s_{2},\theta }^{k}}{\partial d_{k}}\\ \delta_{s_{k1}}=\frac{\partial H}{\partial s_{k1}}=\frac{\partial H}{\partial z_{k}}\frac{\partial z_{k}}{\partial h_{k}}\frac{\partial h_{k}}{\partial \psi_{d,s_{1},s_{2},\theta }^{k}}\frac{\partial \psi_{d,s_{1},s_{2},\theta }^{k}}{\partial s_{k1}}\\ \delta_{s_{k2}}=\frac{\partial H}{\partial s_{k2}}=\frac{\partial H}{\partial z_{k}}\frac{\partial z_{k}}{\partial h_{k}}\frac{\partial h_{k}}{\partial \psi_{d,s_{1},s_{2},\theta }^{k}}\frac{\partial \psi_{d,s_{1},s_{2},\theta }^{k}}{\partial s_{k2}} \end{array}\right.$$

(11) $$\left\{\begin{array}{l} d_{k}=d_{k}-\gamma \delta_{d_{k}}\\ s_{k1}=s_{k1}-\gamma \delta_{s_{k1}}\\ s_{k2}=s_{k2}-\gamma \delta_{s_{k2}} \end{array}\right.$$

Here, ∂ represents the partial derivative, $\psi_{d,s_{1},s_{2,}\theta }^{k}$ represents the third wavelet convolution kernel in AWCM, and $d_{k},s_{k1},s_{k2}$ represents the scaling factor and translation factor, respectively. The parameters are iteratively updated by subtracting the product of the learning rate γ and the gradient δ. Therefore, the iterative updates of the scaling factor d and the translation factor $s_{1},s_{2}$ in the Sym4 wavelet convolution kernel are obtained by substituting $\frac{\partial \psi_{d,s_{1},s_{2},\theta }}{\partial d}$, $\frac{\partial \psi_{d,s_{1},s_{2},\theta }}{\partial s_{1}}$, $\frac{\partial \psi_{d,s_{1},s_{2},\theta }}{\partial s_{2}}$ into the formula.

**Figure 4:**
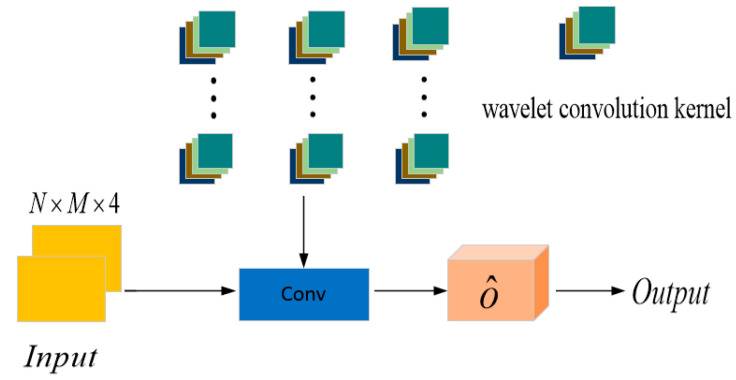
Adaptive wavelet convolution module.

Based on the above substitution derivation, the AWCM is improved by replacing the traditional convolution kernel with an adaptive wavelet convolution kernel, as shown in Figure 4. Due to the self-learning characteristics of the scaling and translation factors, the AWCM module does not introduce a control vector $\hat{o}$ to weight the feature signals.

The selection of the wavelet basis function is critical when constructing an adaptive wavelet convolution module [22], as it determines the network’s initial feature extraction capability and convergence starting point. In this work, the Sym4 wavelet is chosen as the core basis function due to its strong inherent alignment with the characteristics of OTFS signals in the DD domain. The rationale for this selection is analyzed in detail below.

(1) The filter impulse response length of the Sym4 wavelet is 8. Compared to shorter wavelets such as Haar (length = 2) and Db2 (length = 4), the longer filter of Sym4 offers improved frequency localization. This enables more accurate characterization of the complex channel gain of each scatterer in the DD domain of OTFS signals, while avoiding noise amplification caused by excessive localization.
(2) Sym4 possesses a fourth-order vanishing moment. The vanishing moment governs the wavelet’s ability to suppress polynomial components. The equivalent channel response of OTFS in the DD domain exhibits sparse impulse characteristics, with effective information concentrated in a limited number of non-zero taps. The high vanishing moment of Sym4 helps in effectively capturing these dominant components.
(3) The Sym4 wavelet has a continuous second-order derivative, resulting in high regularity in the reconstructed signal. This ensures that the time–frequency feature maps reconstructed from wavelet coefficients are smooth, which aligns well with the continuously varying nature of physical channel responses. This smoothness also helps mitigate potential numerical instability during gradient backpropagation.
(4) Given the OTFS frame dimensions used in this study (N = 8, M = 16), the Sym4 filter with length 8 interacts effectively with the signal grid along both the delay and Doppler axes. Longer filters such as Sym8 may introduce significant boundary effects in small-sized frames, whereas Sym4 strikes a balanced trade-off, allowing the convolution operation to cover meaningful regions without excessively spanning the entire dimension.
(5) Although the Db4 wavelet also possesses fourth-order vanishing moments, its filters are severely asymmetric, which introduces nonlinear phase shifts during signal decomposition and reconstruction, leading to phase distortion in the reconstructed signal. For OTFS systems where symbol phase in the delay-Doppler domain directly conveys modulation information, phase distortion is unacceptable. As a symmetrized variant of the Daubechies wavelet family, Sym4 achieves near-perfect symmetry while retaining fourth-order vanishing moments, thereby suppressing phase distortion to the greatest extent possible. This characteristic grants Sym4 a fundamental advantage over Db4 in phase-sensitive digital communication systems.
(6) Wavelets of the Coiflet family (e.g., Coif4) also support fourth-order vanishing moments but require significantly longer filter support—typically 18 taps for Coif4, compared to only 8 taps for Sym4. Under the OTFS frame dimensions adopted in this study (N = 8, M = 16), excessively long filter support induces severe boundary effects, resulting in incomplete coverage of the delay-Doppler grid along frame edges. By achieving fourth-order vanishing moments with the shortest possible filter length, Sym4 strikes an optimal balance among vanishing moment order, compact support, and boundary effect mitigation.

Based on these considerations, the Sym4 wavelet is selected as the initialization basis function for the adaptive wavelet convolutional layer, providing the network with a high-performance starting point for optimization.

#### 2.2.3. AWCNN-OTFS Network Architecture

**Figure 5:**
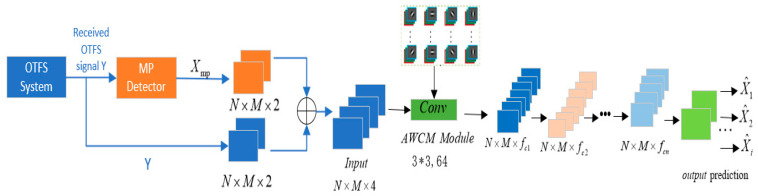
OTFS system signal detection model architecture based on AWCNN.

CNNs are widely used in signal detection due to their powerful feature extraction capabilities. However, the inherent translation invariance of standard convolution operations, along with their limited frequency-domain analysis ability, can become performance bottlenecks when dealing with non-stationary signals or communication signals with specific structures. To overcome this fundamental limitation, this paper proposes an adaptive wavelet convolutional neural network. This method concatenates the preliminary results of MP detection with the original received signal along the channel dimension and replaces the first standard convolution layer with an adaptive wavelet convolution layer to construct a hybrid detection network. This approach enables the network to adaptively learn key features of the signal in the time–frequency domain, thereby achieving better detection accuracy through training and obtaining a detection performance that surpasses CNNs via end-to-end training. The architecture of the AWCNN-based OTFS system signal detection model is shown in Figure 5.

After the input bits pass through the OTFS system, the original received signal Y and the signal $X_{mp}$ obtained through the MP detector are concatenated (“(circle with+)”) along the channel to perform data augmentation and enrich the dataset. MP enhancement further approximates the posterior marginal probability density function of the transmitted signal by continuously passing and exchanging information between the observation nodes and variable nodes in the factor graph model, thereby achieving signal detection. In high-dynamic scenarios, noise can cause error accumulation during MP iterations, manifesting as distortion of the likelihood information. The MP algorithm relies on 10 iterations, with each iteration potentially accumulating errors, leading to an increased proportion of incorrectly decided symbols in hard decision symbols. Additionally, noise peaks may be misjudged as sparse paths, reducing the accuracy of path detection. This paper reduces the traditional 10 MP iterations to 3 and employs an early stopping strategy to suppress noise-induced error accumulation. The hard decision symbols output by MP undergo confidence evaluation, retaining only symbols with posterior probability to generate reliability-enhanced real-valued tensors. Meanwhile, the MP enhancement module utilizes prior knowledge of sparse signal recovery to reduce AWCNN’s dependence on data samples. This intentional design physically severs the path through which errors accumulate and amplify via iterative feedback loops. At this stage, the MP algorithm is not intended to produce final decisions; rather, it generates preliminary soft estimates that encode prior information on inter-symbol correlations. These outputs serve as information-rich feature maps—not hard decisions—thereby preventing the premature solidification of errors. The MP estimates are fed into the AWCNN, but no output from the AWCNN is fed back to the MP. This configuration completely eliminates cross-module iterative loops. Furthermore, the AWCNN itself is a purely feedforward neural network—its detection process is completed in a single forward pass, with no internal iterations or recurrent structures. This ensures that errors cannot circulate or be amplified within the detector. AWCNN employs the ReLU activation function and batch normalization to learn the nonlinear boundary between noise and signal to filter out residual errors. This enables AWCNN to cross-validate and correct MP errors during the learning process. The specific procedure is shown in Algorithm 1.
**Algorithm 1 MP algorithm****Input**: Received signal y, Equivalent channel matrix H
1. Initialize
   $P_{i,j}^{0}=\frac{1}{Q},i=1,\ldots ,NM,j\in J(i)$
   x(c): Calculate the set of observed nodes for each variable node
   y(d): Calculate the set of variable nodes for each observation node
2. **repeat**
3. Observation node y(d) uses the mean $\mu_{i,j}^{n}$ and variance ${\left(\sigma_{i,j}^{n}\right)}^{2}$ of the $P_{i,k}^{n-1}$ interference terms as messages, and passes the messages from the observation node to variable node x(c).
4. Send the message from variable node x(c) to observation node y(c) using $\mu_{i,j}^{n}$, ${\left(\sigma_{i,j}^{n}\right)}^{2}$ and $P_{i,j}^{n-1}$.
5. Use the MAP criterion to make decisions on the output. At the same time, introduce a convergence factor of η to accelerate the algorithm’s convergence.
6. Check the stopping condition: when any one of the three conditions $\eta^{\eta }=1$, $\eta^{\eta }<\eta^{\eta }+\in$, or reaches the set maximum number of iterations, the MP detection algorithm stops iterating and proceeds to the final detection symbol decision.**Output**: $\hat{x}(c)$

This paper designs an adaptive wavelet convolution module (AWCM), with the detailed workflow outlined in Algorithm 2. The core concept of the module is to integrate the time–frequency localization properties of wavelet transform with the adaptive learning capability of convolutional neural networks. By leveraging a learnable Sym4 wavelet basis function [22] to extract multi-scale sparse features from input signals, the AWCM replaces the fixed-kernel feature extraction approach used in conventional convolutional layers. This design enables the multi-level time–frequency information extraction process to be tightly coupled with subsequent classification models. Through backpropagation, all parameters are jointly optimized, allowing the time–frequency features to be dynamically adjusted according to data characteristics during model training, thereby enhancing the robustness and generalization ability of the model.
**Algorithm 2 Adaptive wavelet convolution module****Input:** The original received signal Y and the signal $X_{mp}$ after the MP detector are concatenated along the channel to obtain a new feature tensor $Y_{1}$
Initialize learnable parameters:Lo_D: Low-pass decomposition filterHi_D: High-pass decomposition filter$d_{k},s_{k1},s_{k2}$ represents the scaling factor and translation factor that can be adaptively learned, and the parameters are iteratively updated by subtracting the product of the learning rate and the gradient.Iterate over input channels $c_{in}$Perform convolution operations on each sub-band separately and use learnable Sym4 wavelet filters for feature extractionIterate through all output channels $c_{out}$Upsampling, recombine the processed sub-bands into a complete feature map
**Output:** Feature tensor after adaptive wavelet convolution processing $Y_{2}$


**Figure 6:**
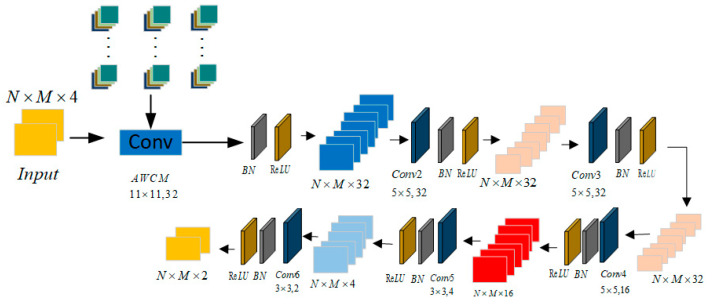
Overall training model for the AWCNN model.

The AWCNN training model proposed in this paper, illustrated in Figure 6, adopts an end-to-end processing pipeline and consists of three main components. First, in the input preprocessing stage, the original signal from the OTFS receiver is concatenated with the preliminary estimates generated by the MP algorithm along the channel dimension. This forms an information-enriched multi-source input tensor, providing enhanced feature information for the network. Subsequently, in the feature extraction stage, the first layer of the conventional convolutional network is replaced with an adaptive wavelet convolutional layer. Leveraging the time–frequency localization properties of the Sym4 wavelet basis function and its learnable scaling and translation factors, this layer is specifically designed to extract sparse features from OTFS signals. The scaling and translation factors are iteratively updated during training according to Equations (10) and (11). This is followed by multiple conventional convolutional layers for further feature learning and training. Finally, in the output module stage, a convolutional layer maps the feature channels to two outputs corresponding to the real and imaginary parts of the signal. A Tanh activation function is then applied to constrain the output values within the range [−1, 1], directly yielding the final estimated transmitted symbols and completing the entire signal detection process. The corresponding loss performance metrics are obtained after training.

The design of this network architecture fully considers the two-dimensional structural characteristics of OTFS signals in the DD domain and the computational efficiency of the model. In VGGNet and its derivative architectures, the shallow convolutional layers commonly adopt 32 channels—a configuration that has been extensively validated across numerous vision and signal processing tasks to offer sufficient feature expressiveness while maintaining a manageable parameter budget. Given the formal similarity between the delay-Doppler representation of OTFS signals and image data, inheriting this well-established design practice is both rational and efficient. The first layer of adaptive wavelet convolution employs an 11 × 11 large convolution kernel with 32 channels, aiming to obtain a broad receptive field at the initial stage to capture the sparse impulses and long-range dependencies of the channel in the DD domain. Subsequently, the network performs deep feature extraction through three consecutive 5 × 5 convolution layers, each with 32 channels. This network design draws inspiration from the successful experience of VGGNet, ensuring nonlinear expressive capability while controlling the number of parameters by stacking small-kernel convolutions. In the deep layers of the network, a “bottleneck” structure is adopted, sequentially reducing the number of channels to 16 and 4 using 3 × 3 convolutions. This approach aims to compress features and enhance the model’s generalization ability. Finally, the output layer uses a 3 × 3 convolution kernel to produce 2 channels, with the Tanh activation function employed to directly correspond its output to the normalized constellation range of modulation symbols.

The selection of these hyperparameters is determined based on theoretical guidance and preliminary architecture search experiments, achieving a good balance between performance and complexity on the validation set. Although there is potential to further explore performance through automated hyperparameter optimization techniques, the core objective of this paper is to verify the effectiveness of the AWCM; the current network structure has provided a solid foundation for fair comparisons.

### 2.3. Model Training

The random bits are passed through the OTFS system to generate OTFS signals, which are then input into the MP detector to obtain a new detected signal $X_{mp}$. This is concatenated with the received OTFS signal Y along the channel to form a new feature signal $Y_{1}$. The signal dataset is then input into the AWCNN for training. The adaptive learning scaling factor and translation factor parameters in AWCM are updated using the above Formula (11); other trainable parameters in the model are updated using the Adam backpropagation algorithm. Finally, the test set in the dataset is input into the corresponding network model for performance testing to obtain its evaluation metrics. The trainable parameters θ of the AWCNN network serve as features, mapping the high-dimensional fused tensor $Y_{1}$ to the estimated transmitted symbols $\hat{X}$. The learning function is given in Formula (12):

(12) $$\hat{X}\to f(Y_{1};\theta )$$

Using the training dataset, the network parameters θ are optimized by minimizing the average loss. Under a certain signal-to-noise ratio, the training sequences known to both the transmitter and receiver, along with their outputs through the doubly extended channel, are used as the training set for the AWCNN model. The output layer uses a regression function, then the loss function is minimized using the mean squared error function, as shown in Equation (13):

(13) $$Loss=\frac{1}{D}\sum_{d=1}^{D}{\left(t_{d}-y_{d}\right)}^{2}$$

In the formula: $t_{d}$ and $y_{d}$ refer to the network-predicted target value and output, respectively.

The AWCNN learning parameter update algorithm uses the Adam algorithm, where the learnable parameters include: the wavelet basis function parameters of the adaptive wavelet convolution layer, the weights and biases of subsequent convolution layers, the parameters of the batch normalization layers, and so on.

## 3. Results

### 3.1. Bit Error Rate Performance Analysis

In order to evaluate the performance of the AWCNN-based OTFS system signal detection method proposed in this paper, this section simulates and obtains performance metrics using PyCharm Community Edition 2020.1.3×64 Software, comparing them with traditional convolutional neural networks. The OTFS system uses the QPSK modulation scheme, with the number of symbols N being eight and the number of subcarriers M being 16. The carrier frequency $f_{c}$ is 2 kHz and the subcarrier spacing Δf is 15 GHz. This channel model only considers tapped channels under integer multiples of Doppler shifts and delays. The channel coefficients of different paths are generated using a complex Gaussian random distribution with a mean of 0 and a variance of 1/3. Each channel coefficient is independent and identically distributed. The bitstream of each transmitted frame is generated randomly. During the simulation, it is assumed that the receiver knows the channel matrix H. The detailed simulation parameters of the OTFS system are shown in Table 1.

The network construction and training are implemented in PyCharm Community Edition 2020.1.3×64; the designed AWCNN detector model is shown in Table 2.

This simulation experiment generates a dataset [23] of 1000 frames based on the OTFS system parameters in Table 1 and transmits it through a delay-tap channel. The data is divided into a training set of 700 frames, a validation set of 150 frames, and a test set of 150 frames. The AWCNN model is shown in Table 2 and is implemented using a custom learning framework in PyCharm Community Edition 2020.1.3×64, utilizing the Adam optimizer with an initial learning rate of 0.009, a batch size of 32, and a maximum of 64 training epochs. The model input is a four-channel tensor. The first layer is an adaptive wavelet convolution layer, which learns the scaling and translation parameters of the Sym4 wavelet basis adaptively. This is followed by five conventional convolutional layers (32 → 32 → 32 → 16 → 4 channels, all using 5 × 5 kernels with padding = 2) and two convolutional layers (4 → 2 channels, 3 × 3 kernels with padding = 1). Each convolutional layer is followed by batch normalization (BN) and a ReLU activation function. The model outputs a two-channel feature map, which after activation produces a normalized two-channel tensor. Finally, the output layer uses the Tanh function to achieve accurate symbol detection through optimal decision-making.

**Figure 7:**
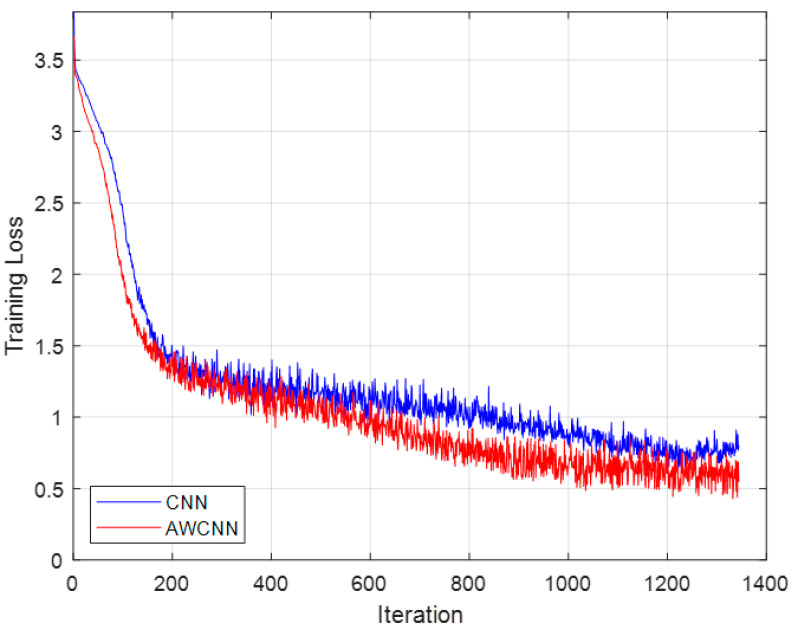
Convergence curves of two models under QPSK modulation in OTFS system.

The dataset used in this study consists of 1000 OTFS signal frames. This configuration is chosen to strike a balance between experimental efficiency and proof-of-concept validity during the initial exploratory phase of method development. As shown in Figure 7, the training and validation losses decrease synchronously and plateau without significant divergence, indicating that no overfitting occurs. Moreover, the model achieves a consistently low bit error rate (BER) on the 150-frame test set, which is completely unseen during training. This result directly substantiates the generalization capability of the proposed method under the current dataset configuration. Figure 7’s simulation results show that the convergence curves of the AWCNN model proposed in this paper are consistently better than those of the CNN model. Specifically, AWCNN can reach the loss level achieved by the CNN model after convergence with fewer training epochs, which confirms that the adaptive wavelet convolution module effectively accelerates model training by enhancing the ability to extract the sparse features of OTFS signals, resulting in superior convergence performance.

**Figure 8:**
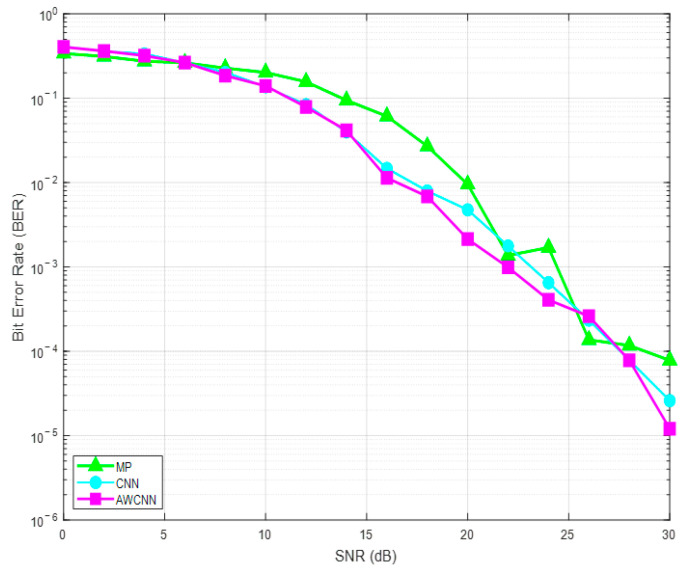
BER performance curves of different detection algorithms.

Figure 8’s experimental simulation shows that the proposed AWCNN method effectively addresses the issue of insufficient feature extraction in CNN models. In terms of performance, this method can achieve a bit error rate comparable to the CNN algorithm under a low signal-to-noise ratio of 2 dB. In addition, this method does not require pilot assistance to obtain channel state information, significantly reducing system overhead.

In this simulation experiment, the three-tap channel model in Table 1 is replaced with the EVA channel model defined by the 3GPP standard. Compared to the equal-gain three-tap delay channel model, the EVA model includes nine non-uniform power delay taps, with a maximum delay spread of 2510 ns and a maximum Doppler shift of approximately 289 Hz at a simulated speed of 120 km/h. This channel model can more realistically reflect the frequency-selective fading and time-varying characteristics in high-speed mobile scenarios. The specific parameters of the EVA channel are shown in Table 3.

**Figure 9:**
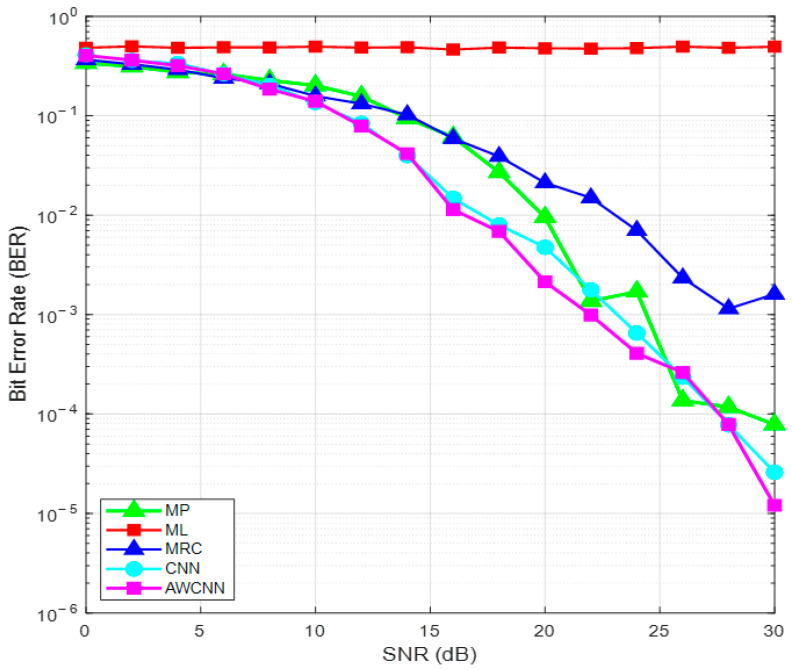
BER performance curves of different detection algorithms.

In order to comprehensively evaluate the performance advantages of the AWCNN method, this section compares the proposed method with the existing detection algorithms mentioned in Chapter 3 under the EVA channel. Figure 9 shows a comparison of the BER performance of the proposed AWCNN and the existing detection algorithms from 0 to 30 dB.

Overall, at medium-to-high SNR, the proposed AWCNN method demonstrates significantly superior BER performance compared to benchmark algorithms such as ML, MRC, MP, and CNN.

Specifically, the ML algorithm exhibits the poorest performance, followed by MRC. The MP algorithm outperforms MRC, and while CNN shows improvement over MP, it remains inferior to the proposed AWCNN. The superior performance of AWCNN is primarily attributed to its use of adaptive wavelet convolutional kernels, which replace the traditional fixed kernels. This design leverages the time–frequency localization properties of the Sym4 wavelet basis and enhances the ability to extract sparse features from OTFS signals through learnable scaling and translation parameters.

Experimental simulations indicate that at an identical BER, AWCNN achieves an approximate 2 dB SNR gain over the standard CNN algorithm while also exhibiting faster training convergence. Furthermore, this method accomplishes detection without requiring pilot-assisted channel state information acquisition.

### 3.2. Computational Complexity Analysis

In order to comprehensively evaluate the AWCNN method, this section provides a theoretical analysis of the computational complexity of various detection algorithms. Table 4 presents a comparison of the computational complexity of different algorithms when processing a single-frame OTFS signal.

Computational complexity analysis reveals significant trade-offs between performance and computational overhead across different detection algorithms. While the ML detector theoretically achieves optimal performance, its exponential computational complexity makes it impractical for real-time communication scenarios. The MRC algorithm maintains an advantage with the lowest linear complexity but suffers from inherent limitations in detection performance. Although the traditional MP algorithm outperforms MRC, it requires extensive iterative operations to ensure accuracy.

First, the performance gap dictates the difference in benchmarks. MRC and similar linear detectors are fundamentally incapable of handling the severe ICI induced by Doppler spread in OTFS systems under high-mobility scenarios. Their BER performance deteriorates rapidly to an unusable level in high-Doppler channels. Therefore, AWCNN addresses a detection problem characterized by strong nonlinear interference—a task for which MRC is inherently unsuitable. The increased computational overhead of AWCNN is exchanged for a fundamental leap in capability: from being “essentially inoperable” to achieving “reliable communication.” This trade-off is not only necessary but also justified for any practical high-mobility communication system. Second, compared to the high-performance MP detector, AWCNN offers superior deterministic efficiency. While the traditional message-passing algorithm achieves good performance by iteratively exploiting channel sparsity, its computational cost grows linearly with the number of iterations. Moreover, the iterations required for convergence vary dynamically with channel conditions, which is detrimental to ensuring determinism and low latency in hardware implementation. In contrast, AWCNN replaces the uncertain, multi-iteration process of the MP algorithm with a single, deterministic forward pass. This approach provides fixed and predictable processing latency while delivering comparable or superior BER performance, which is crucial for real-time communication systems.

Compared to the basic CNN architecture, the proposed AWCNN method introduces additional computational overhead due to the incorporation of adaptive wavelet decomposition and message-passing enhancement modules. However, experimental results demonstrate that this moderate increase in complexity yields approximately 2 dB of SNR gain, which is justified from a system design perspective. This performance improvement translates into significantly reduced transmission power requirements, and in energy-constrained application scenarios, this advantage sufficiently compensates for the increased computational costs.

## 4. Discussion

While the proposed AWCNN demonstrates excellent detection performance under the custom doubly selective channel model, this study acknowledges certain limitations in channel modeling. The current evaluation primarily employs a simplified delay-tap channel model to validate this approach, which may not fully capture the complexities of real-world deployment scenarios. Particularly, the performance under more realistic standardized channel models—such as the 3GPP Extended Vehicular A (EVA) and Extended Typical Urban (ETU) models that incorporate fractional Doppler shifts and richer multipath characteristics—remains to be systematically investigated in future work.

However, we fully recognize that in real-world high-mobility communication scenarios, channels often exhibit fractional Doppler shifts and delays. This non-grid-like path characteristic disrupts the strict sparsity of the delay-Doppler domain channel, leading to energy dispersion and exacerbated intercarrier interference, which is widely regarded as one of the core challenges for the practical deployment of OTFS systems.

It is noteworthy that the design mechanism of the AWCNN method proposed in this paper offers unique potential to address this challenge. The adaptive wavelet convolutional layer, through its learnable scaling and translation factors, can theoretically dynamically adjust the time–frequency focusing region of its basis functions. This enables it to learn and match the energy dispersion profile caused by fractional effects, thereby achieving a data-driven interference suppression mechanism. Furthermore, the symbol correlation priors provided by the integrated message-passing preprocessing module also assist the network in making distinctions within more complex interference environments.

Regarding the scalability of the proposed method to larger OTFS frame sizes (beyond the tested configuration of N = 8 and M = 16), we elaborate on two key aspects: architectural design and adaptive mechanism.

First, the proposed AWCNN is a fully convolutional network without any fully connected layers. The spatial dimensions of its output feature maps are dynamically determined by the input size. By adopting a padding = ‘same’ strategy, the network can naturally accommodate input tensors of varying dimensions without structural modification. Moreover, due to parameter sharing in convolutional layers, the computational complexity scales linearly with the input size, while the total number of parameters remains constant. This ensures favorable computational scalability.

Second, the core module—AWCM—possesses intrinsic parametric adaptability. Its learnable scaling and translation factors are automatically optimized according to the data distribution during training. As the frame size expands and the sparse feature distribution in the delay-Doppler domain evolves, the adaptive wavelet kernels can adjust their time–frequency-focusing regions through training to match the extended or denser feature patterns. Furthermore, the inherent multi-resolution analysis (MRA) capability of wavelet transforms enables natural adaptation to channel feature extraction across different scales, offering superior generalization ability compared to fixed-size standard convolutional kernels.

In summary, the proposed method demonstrates strong scalability potential at both the architectural level and the core module level. Due to space limitations and experimental scope, quantitative validation on larger OTFS frame sizes is left for future work.

## 5. Conclusions

This paper addresses the challenging problem of signal detection in doubly dispersive channels for OTFS systems operating in high-mobility scenarios by proposing a novel approach based on an adaptive wavelet convolutional neural network. The proposed method effectively mitigates the issues of slow convergence and high computational costs associated with conventional CNNs in detecting OTFS signals over such channels. By integrating message-passing (MP) algorithms with data augmentation using raw received signals, the method enriches the input feature representation. Leveraging the time–frequency localization properties of Sym4 wavelets and adaptive parameter learning, it achieves efficient extraction of sparse signal characteristics. Experimental results demonstrate significant improvements in both training loss and training time.

Although the proposed AWCNN-based detection method exhibits superior performance under ideal integer DD channel conditions, its practical applicability requires further validation in more complex channel environments. Future research will focus on extending the method to more realistic scenarios, including fractional delay-Doppler channels and standardized 3GPP channel models. This evolution aims to transform the method from a specialized detector for ideal channels into a versatile solution capable of operating in real-world complex propagation environments, thereby laying a solid theoretical and technical foundation for 6G high-mobility communication systems.

## 6. Patents

«A detection method for OTFS signals based on an adaptive wavelet scattering con-volutional neural network» Patent number: 202510020729.3.

## Figures and Tables

**Table 1:** OTFS system simulation parameters.

Parameter Name	Parameter Value
OTFS frame size (N, M)	(8, 16)
Subcarrier interval/kHz	240
Carrier frequency/GHz	15
Bandwidth/kHz	2
Modulation method	QPSK
Channel path taps	3
delay_ taps delay_ taps/μs	[0, 5, 10]
Doppler frequency bias _ taps/Hz	[0, 1, −1]
SNR/dB	0:2:30

**Table 2:** AWCNN detector parameters.

Layer	Parameters	Activation Function
Input layer	N × M × 4	-
AWCNN Layer1	(11 × 11.32)	ReLU
Convolutional Layer2	(5 × 5.32)	ReLU
Convolutional Layer3	(5 × 5.32)	ReLU
Convolutional Layer4	(5 × 5.32)	ReLU
Convolutional Layer5	(3 × 3.16)	ReLU
Convolutional Layer6	(3 × 3.4)	ReLU
Output layer	(3 × 3.2)	Tanh

**Table 3:** EVA channel simulation parameters.

Tip Number	Multipath Delay/ns	Normalized Power/dB
1	0	0.0
2	30	−1.5
3	150	−1.4
4	310	−3.6
5	370	−0.6
6	710	−9.1
7	1090	−7.0
8	1730	−12.0
9	2510	−16.9

**Table 4:** Comparison of the computational complexity of different detection algorithms.

Detection Algorithm	Computational Complexity	Explanation of Key Parameters
ML	$O(TH^{2})$	Time step T, hidden layer H
MRC	O(NK)	Number of antennas N, number of symbols K
MP	O(IPK)	Number of iterations (I = 10), number of paths (P), number of symbols (K)
CNN	$\begin{array}{l} O(D\times (C\_in\times C\_out\times F\_h\times F\_w)\\ \times H\times W) \end{array}$	CNN depth (D), F_h/F_w: kernel size, H/W: feature map size
AWCNN	$\begin{array}{l} O(D\times (C\_in\times C\_out\times F\_h\times F\_w)\\ \times H\times W)+O(C\_in\times C\_out\times S) \end{array}$	S: the scaling and translation parameter space of the wavelet convolution kernel

## Data Availability

During the research period, links to publicly archived datasets that were analyzed or generated are displayed at: Zhou, M. “OTFS AWCNNdataset”, Mendeley Data, V1. 2025. Available online: https://data.mendeley.com/datasets/gp5hht8gys/1 (accessed on 12 November 2025).
